# Four new West Palaearctic species and new distributional records of Hybotidae (Diptera)

**DOI:** 10.3897/zookeys.1019.61496

**Published:** 2021-02-22

**Authors:** Liliana Kanavalová, Patrick Grootaert, Štěpán Kubík, Miroslav Barták

**Affiliations:** 1 Department of Zoology and Fisheries, Faculty of Agrobiology, Food and Natural Resources, Czech University of Life Sciences Prague, Kamýcká 129, 165 00, Praha-Suchdol, Czech Republic Czech University of Life Sciences Prague Czech Republic; 2 Department of Entomology, Royal Belgian Institute of Natural Sciences, Rue Vautier 29, B – 1000, Brussels, Belgium Department of Entomology, Royal Belgian Institute of Natural Sciences Brussels Belgium

**Keywords:** Descriptions, distribution, *
Hybos
*, *
Megagrapha
*, new species, *
Oedalea
*, *
Platypalpus
*, re-description, *
Syndyas
*, taxonomy

## Abstract

*Megagrapha starki* Barták & Grootaert, **sp. nov.** (Poland, Russia, Slovakia), *Oedalea portugalica* Barták & Grootaert, **sp. nov.** (Portugal), *Hybos conicus* Grootaert & Barták, **sp. nov.** (Greece, Turkey), and *Platypalpus obscuroides* Barták & Grootaert, **sp. nov.** (Slovakia) are described and illustrated. Diagnostic characters are discussed. The female of *Syndyas merzi* Shamshev & Grootaert, 2012 is described for the first time. New distributional records are presented: *Megagrapha europaea* Papp & Földvári, 2001 is first reported from Slovakia and *Syndyas merzi* Shamshev & Grootaert, 2012 is first reported from Turkey.

## Introduction

The family Hybotidae comprises more than 2000 described species distributed worldwide (Yang et al. 2007) in 75 genera (Pape et al. 2011). Several recent papers have tried to elucidate the phylogeny, taxonomy and natural history of the Hybotidae (Chvála 1983; Wiegmann et al. 1993; Collins and Wiegmann 2002; Moulton and Wiegmann 2004, 2007; Sinclair and Cumming 2006; Nagy et al. 2013; Wahlberg and Johanson 2018).

Hybotids are yellow to black flies, small (mostly 1–7 mm), and the vast majority of known species are predators and are usually found on vegetation, logs, stones and other surfaces (Barták and Kubík 2018). Despite extensive research on this family in the West Palaearctic, and especially Europe, still many new species have been recently described, for example from the genera *Bicellaria* (Barták and Kubík 2013), *Chersodromia* (Grootaert et al. 2010, 2012b; Grootaert and Shamshev 2010), *Drapetis* (Michelsen and Grootaert 2019), *Euthyneura* (Shamshev and Kustov 2012), *Hybos* (Shamshev et al. 2015), *Platypalpus* (Grootaert 2008; Grootaert et al. 2012a, 2020; Kustov et al. 2014, 2015; Barták and Kubík 2015, 2016, 2018; Grootaert and Alexiou 2020), *Syndyas* (Shamshev and Grootaert 2012), *Tachydromia* (Gonçalves et al. 2021) and *Trichina* (Barták and Kubík 2009). Moreover, the diversity of hybotids has probably been greatly underestimated in several regions, e.g. in Central Africa and the Oriental and Neotropical Regions (Nagy et al. 2013). Our present paper aims to add some knowledge concerning the West Palaearctic. In the light of the unprecedented reduction of biodiversity and the possible further mass extinction of biota we should preserve species at least in collections for further studies (Barták 2019).

## Material and methods

The material studied originated from recent collections of two of the authors (M. Barták and Š. Kubík) in Spain, Portugal and Turkey and our colleagues Michal Tkoč (National Museum, Praha), Jindřich Roháček (Silesian Museum, Opava), and Jan Ševčík (University of Ostrava, Ostrava) in Slovakia (even though only one of them was listed on locality labels, they all together collected materials and operated Malaise traps). The material is deposited in the collection of the Czech University of Life Sciences, Prague (**CULSP**), and partly in the Royal Belgian Institute of Natural Sciences (**RBINS**), the Zoological Museum of Moscow University (**ZMMU**), and the private collection of A. Stark (**CAStH**). The material from Spain, Portugal and Turkey was collected by means of mass trapping methods (sweeping vegetation [SW], yellow and white water pan traps [PT], Malaise traps [MT]) and stored in ethyl alcohol; material from Slovakia was collected by means of Malaise traps. Voucher specimens were selected and dried using the method described by Barták (1997).

Genitalia preparations and drawings: the male terminalia were removed from the body, softened in tap water for 10 min and transferred to 10% KOH in a plastic tube with a screw-on stopper. The tube was submerged in hot water (at least 60 °C) in a thermos bottle for about 1 hour and the state of maceration was verified regularly. When all darkly sclerotized structures were transparent enough, the terminalia were rinsed in tap water for about 5 min, transferred to 70% ethanol for 10 min and transferred to pure glycerine. The glycerine -soaked terminalia were finally mounted in a neutral gel, orientated in the required position, and drawn with a camera lucida mounted on a Leitz Laborlux microscope.

The morphological terms used here follow Cumming et al. (1995), Merz and Haenni (2000), Sinclair (2000), and Sinclair and Cumming (2006). All body measurements (including body and setae length) were taken from dry specimens (therefore the actual length may differ from that of fresh or wet-preserved material) by means of an ocular micrometer mounted on a Nikon SMZ 1500 binocular microscope. Male body length was measured from the antennal base to the tip of the terminalia and female body length from the antennal base to the tip of the cerci. Thoracic setae were counted on one side of body.

## Taxonomy

### Descriptions of new species

#### Hybos conicus

Taxon classificationAnimaliaDipteraHybotidae

Grootaert & Barták sp. nov.

AB0D28D2-C6F0-58D3-9001-28A39CB60670

http://zoobank.org/E68B526B-FEB4-4FA2-B64A-F36CF5FB4DB4

Figs 1 , 2

Hybos culiciformis (Fabricius, 1775): partim in Shamshev et al. (2015: 455, figs 10, 15, 20).
Hybos sp. nr. culiciformis (Fabricius): Barták and Kubík (2018: 478).

##### Type material.

***Holotype*** ♂, **Turkey**, Akyaka, forest, 30 m, 37°03'16"N, 28°19'35"E, Barták, Kubík, 30.iv.–9.v.2013 (CULSP). ***Paratypes***: 3♂, same data as holotype; 1♂, 2♀, same locality, 40 m, SW, 37°03'19"N, 28°19'36"E, Barták, Kubík, 26.iv.2016; 1♂, Akyaka, pasture, 8 m, 37°03'11"N, 28°20'33"E, Barták, Kubík, 27.iv.2016; 5♂, 11 km E of Muğla, pine wood + meadow, 1310 m, 37°12'45"N, 28°27'42"E, Barták, Kubík, 1.v.2013; 1♂, 2♀, 13 km NE of Muğla, pasture/pine wood, 1200 m, 37°14'50"N, 28°30'E, Barták, Kubík, 23–27.vi.2015; 1♂, 1♀, same locality, 37°15'N, 28°30'E, 1100–1300 m, Barták, Kubík, 2–3.v.2016; 2♀, Muğla University campus, edge of pine wood + *Quercus* shrubs, 710 m, MT, 37°09'39"N, 28°22'20"E, Barták, Kubík, xi.2012–iii.2013; 1♂, same locality, 37°09'42"N, 28°22'13"E, H. Kavak, 26.v.–26.vi.2015; 4♂, 3♀, Gökçeova Gölü, lake shore, 1750 m, 37°03'42.52"N, 28°48'28.42"E, Barták, Kubík, 20.ix.2012; 1♂, 8 km S of Çine, river bank, 68 m, SW, 37°32'34"N, 28°03'46"E, Barták, Kubík, 29.iv.–1.v.2016; 5♂, 1♀, **Greece**: 7 km E of Mt. Olympus, wood, excrements, 1100 m, 40°6'13"N, 22°25'36"E, M. Barták, 23.v.2007 (all CULSP, except 1 ♂ RBINS).

##### Diagnosis.

A species of *Hybos* Meigen very similar to *H. culiciformis*, but with postpedicel longer than bare apical mechanoreceptor, dark scutellars, shorter ventral preapical seta on mid tibia, and lateral margins of tergites 3–5 almost entirely microtrichose. The most important difference is in the male terminalia: the right epandrial lamella extends posteriorly into a cone-shaped projection.

##### Etymology.

The species is named after the conical right epandrial lamella.

**Figure 1.**
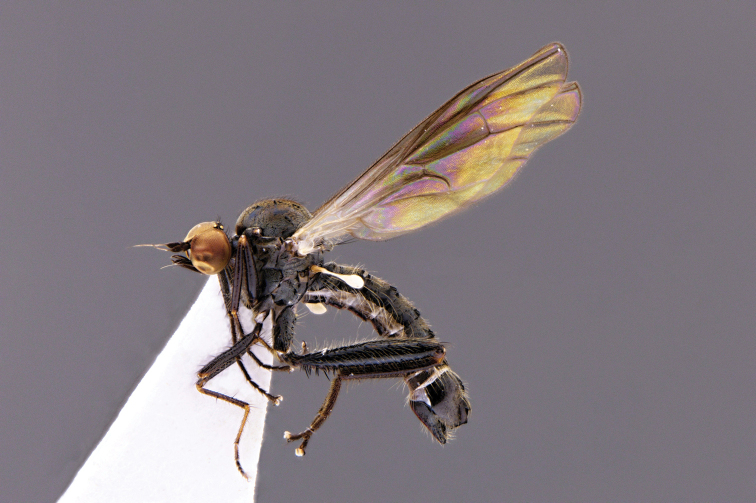
*Hybos conicus* sp. nov., male habitus, paratype.

##### Description.

**Figure 2.**
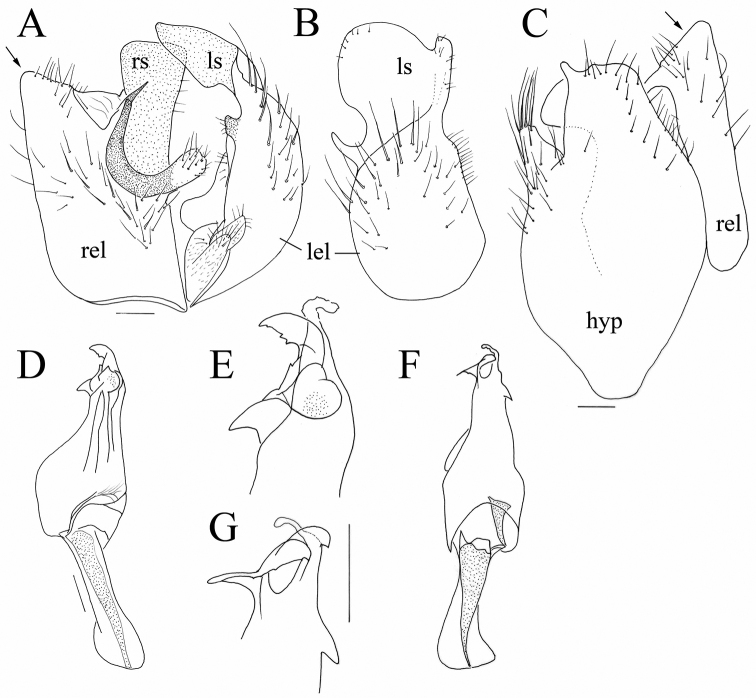
*Hybos conicus* sp. nov. Male terminalia **A** epandrium, dorsal view **B** left epandrial lamella **C** hypandrium **D** phallus, lateral view **E** phallus tip, lateral view **F** phallus, ventral view **G** phallus, detail of tip, ventral view. Abbr. hyp: hypandrium; lel: left epandrial lamella; ls: left surstylus; rel: right epandrial lamella; rs: right surstylus. Arrow indicates conical apex of right epandrial lamella. Scale bars: 0.1 mm.

***Male head*** black, holoptic. Eyes contiguous over long distance leaving only very small triangle above antennal base, upper ommatidia much larger than lower. Prominent and microtrichose ocellar triangle with pair of black setae (about 0.18 mm long) and additional pair of much smaller setae posteriorly. Occiput microtrichose, with black setae on dorsal third subequal to length of ocellars, arranged almost in single row (with only several setae posteriorly), which moves away from eye margin towards middle of occiput, being ventrally replaced by another postocular row of setae starting at about middle of eye hind margin. Face microtrichose, rather narrow (0.07 mm at middle, narrowing ventrally), clypeus lustrous, gena invisible. Palpus brown, narrow basally, slightly broadened apically, reaching tip of proboscis, with several short black setae. Proboscis brownish-black, shiny, directed slightly obliquely anteriorly, about 0.60 mm long (subequal to head length), ventrally with several very short spines. Antenna black, scape (0.04–0.05 mm long) without setae, pedicel (0.05–0.07 mm) with circlet of short setae. Postpedicel elongate ovate (0.17–0.21 mm long and 0.08–0.09 mm wide), stylus whitish (darker basally), more than twice longer than postpedicel (0.38–0.45 mm long), with rather long, bare apical mechanoreceptor (0.10–0.15 mm long). ***Thorax*** moderately arched, black, entirely microtrichose (microtrichia rather long), except small lustrous spots on sides of antepronotum behind setae and shiny apex of postpronotum. Scutum (especially central part) with distinct brownish tomentum, posthumeral area with “vortex” of microtrichia giving impression of dark spot from anterodorsal view. Chaetotaxy: prosternum (isolated sclerite) without setae, antepronotum with collar of short black setae, postpronotal seta small, presutural intra- and supra-alar not differentiated from setulae, acrostichals irregularly 6-serial, short (about 0.10 mm), only narrowly separated from dorsocentrals (rarely acrostichals almost 4-serial, sometimes with several setae inserted in interspace between outer acrostichal row and dorsocentrals). Dorsocentrals irregularly uniserial, subequally long as acrostichals or slightly longer, ending in one strong black seta inserted a long distance from scutellum. Single long black to pale proepisternal seta, usually 2 long, strong black notopleurals (lower one half as long as upper one), 1 postalar, scutellum with pair of usually black (rarely pale), long strong setae and additional much smaller pale setae (usually 4 pairs). ***Legs***: coxae black, microtrichose (except shiny spot anteriorly on hind coxa) and pale setose. Femora black, fore and hind tibiae often lighter (reddish-brown), mid tibia yellow to reddish-yellow (colour of tibiae rather variable), tarsi usually brown, knees of all legs yellowish. Coxae mostly pale setose, legs with both pale and black setae. Fore femur and tibia slightly broader than mid femur and tibia. Fore femur with sparse setae ventrally, shorter than femur depth. Fore tibia with one strong, submedian anterodorsal seta and a similarly long, preapical anterior seta, other setae short, posterior and posteroventral setulae scarcely longer than tibia depth; similar setae on very narrow and long fore tarsus. Mid femur with row of several anterodorsal black setae shorter than femur depth, ventral setosity similar to fore femur. Mid tibia with 2–3 anterodorsals and 1–3 anteroventrals, preapical anteroventral (or ventral) seta about 0.20–0.30 mm long; mid tarsus similar to fore tarsus, basitarsus only slightly shorter. Hind femur swollen, ventral spines in proximal third of femur arranged in 2–3 irregular rows, in more distal part anteroventral row consists of 4–6 longer spines (about 0.15 mm long) and posteroventral row of much shorter and more densely arranged spines, area posteriorly of posteroventral spines with rather long, mostly pale posteroventrals slightly longer than femur depth in apical third, with several spine-like anterodorsals slightly longer than femur depth. Hind tibia slender, with thin setulae at most slightly longer than tibia depth; tarsus slightly shorter than fore tarsus, basitarsus ventrally with very short spines. ***Wing*** darkened to various extent, some specimens (immature?) with almost hyaline wings, but others with wing distinctly darkened, darker in area proximal of basal crossveins and anteriorly (in radial cells). Pterostigma almost hyaline in light winged specimens but dark brown in specimens with darkened wing, elongate-ovate, symmetrically around tip of vein R_1_. Wing entirely microtrichose; basal costal seta absent; Sc incomplete, apically closely approaching R_1_. Costa ends at tip of M vein, anal vein complete and depigmented. Halter pale yellow, calypter whitish-yellow with white margin and yellow fringes. ***Abdomen*** nearly entirely microtrichose, tergites with only narrow hind margin lustrous (shiny margin occupies less than one-third of length of tergites), whole abdomen pale (yellow to white) setose. Dorsum of tergites with very short setulae, lateral parts with setae subequally as long as their segments (longest on segments 2–3, up to 0.35 mm long). Sternites 1–2(3) shiny, remaining sternites microtrichose with shiny posterior part, sparsely setose with rather long pale setae, sternite 1 bare. Male terminalia as in Figure 2. Right epandrial lamella with conical tip (Fig. 2A, C, arrow), bearing rather short setae. Right surstylus saddle-shaped with truncate tip. Left surstylus as in Fig. 2B. Apex of hypandrium truncate with short finger-like projection towards left side (Fig. 2C). ***Length***: body 3.1–4.4 mm, wing 3.1–4.1 mm.

**Female.** Very similar to male except usual sexual differences. Abdomen similarly coloured and setose as in male; tergites with narrower hind lustrous margin (occupying about 1/4 of tergite length), tergite 7 encircling almost entire abdomen, similarly setose as preceding one, sternite 7 widened, broader than long; tergite 8 dorso-apically depigmented, laterally with long darkened setae overreaching abdomen (longest about 0.30 mm long), sternite 8 heart-like, widened, posteromedially with U-shaped cavity bearing 2 long, close-set spine-like setae directed dorsally. ***Length***: body 3.4–4.4 mm, wing 3.4–4.4 mm.

##### Remarks.

The Palaearctic species of *Hybos* were recently treated by Shamshev et al. (2015). In that paper it was shown that one of the most common European species, *Hybos culiciformis* (Fabricius, 1775), consisted of a complex of populations with a gradient of differences in the male terminalia and the colour of setae. The male terminalia of the Mediterranean species probably form a geographic gradient and since the differences were not supported by COI barcoding (Shamshev et al. 2015), these different “populations” were not split up into species. However, we now consider one species as distinctly different (differences outlined below) and we treat it as a separate species described here as new (*Hybos conicus* sp. nov., HCO). HCO is very similar to *H. culiciformis* (HCU), however, HCO has a larger postpedicel (longer than bare apical mechanoreceptor, shorter in HCU), dark scutellars (mostly yellow in HCU), shorter ventral preapical seta on mid tibia (usually shorter than 0.35 mm in HCO but longer than 0.40 mm in HCU) and larger microtrichose areas of the abdomen (lateral margins of tergites 3–5 almost entirely microtrichose in HCO, but almost entirely lustrous in HCU). The most important difference is in the male terminalia: the right epandrial lamella extends posteriorly into a cone-shaped projection in HCO, and is easily visible without dissection.

The new species was previously illustrated in Shamshev et al. (2015) on the basis of a male from Greece. The cone-shaped right epandrial lamella is similar to Shamshev et al. (2015, fig. 10) versus Fig. 2A as well as the shape and bristling of the left epandrial lamella in Shamshev et al. (2015, fig. 15) versus Fig. 2B and the hypandrium in Shamshev et al. (2015, fig. 20) versus Fig. 2 C. The side of the phallus does not bear spinules like in specimens of true *H. culiciformis* (Shamshev et al. 2015, figs 5, 6 versus Fig. 2D of *H. conicus* sp. nov.).

Raffone (2011) described *H. fulvitarsatus* Raffone and the differences from *H. culiciformis* stated in that paper are all highly variable; moreover, illustrations of the terminalia clearly correspond the *H. culiciformis*. *H. fulvitarsatus* is apparently different from the above-described species and it is probably identical to the *H. culiciformis*, but a formal synonymy will be possible only after the examination of type specimens.

##### Distribution.

Turkey, Greece.

#### Megagrapha starki

Taxon classificationAnimaliaDipteraHybotidae

Barták & Grootaert sp. nov.

80E40E77-44EF-5E21-A3D1-235F0EEC16C8

http://zoobank.org/0023393E-CB52-4C09-A30D-4D1AC3D3D336

Figs 3 , 4 , 5 , 6

##### Type material.

***Holotype*** ♂, **Slovakia**: Muráňska planina NPR, Šarkanica, 540 m, MT, 48°42'46"N, 19°59'52"E, M. Tkoč, 26.iv.–15.vi.2017 (CULSP). ***Paratypes*: Slovakia**, 1♂, Muráňska planina NP, 580 m, MT, 48°42'46"N, 19°59'56"E, J. Roháček, 14.vi.–11.vii.2017 (dissect., RBINS); **Poland**: 1♂, Bialowieza, ‘Bialowieza Forest’, Promotion Area, ‘Lesna’ Reserve, 15 km SE of core zone of NP, 160 m a.s.l., 120 year old Carpinus betulus fogged with pyrethrum, 52°37'41"N, 23°46'12"E, A. Floren, 28.vi. 2001 (CAStH); **Russia**: 1♂, SW Moscow, Moskovskiy, forest edge, YPT traps, 55°35'16"N, 37°19'58"E, K. Tomkovich, 1–4.viii.2014 (ZMMU).

##### Diagnosis.

**Figure 3.**
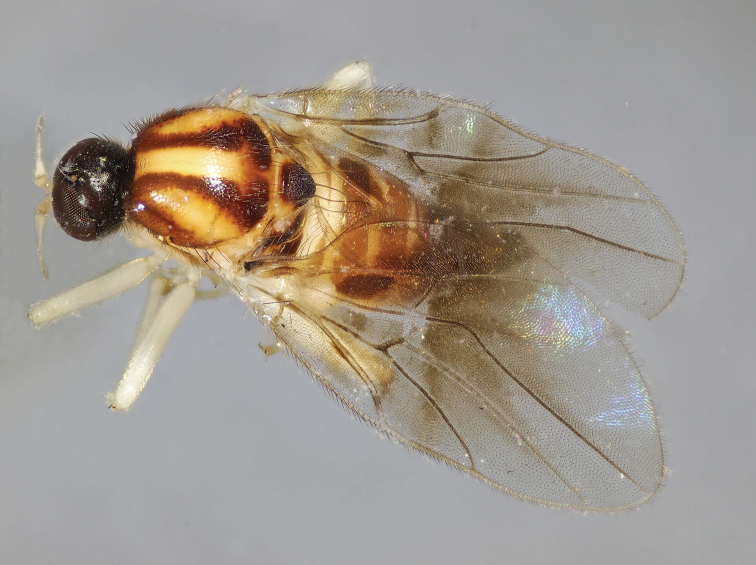
*Megagrapha starki* sp. nov. Male habitus, paratype.

**Figure 4.**
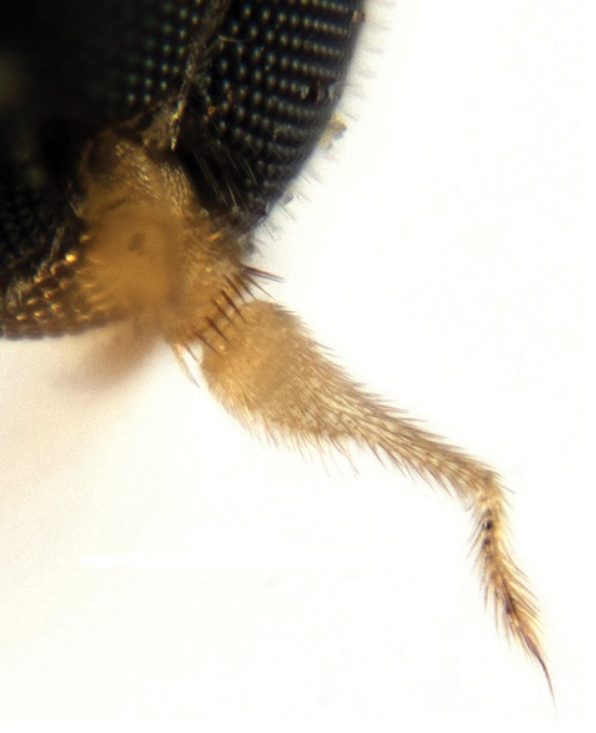
*Megagrapha starki* sp. nov., male antenna, paratype.

**Figure 5.**
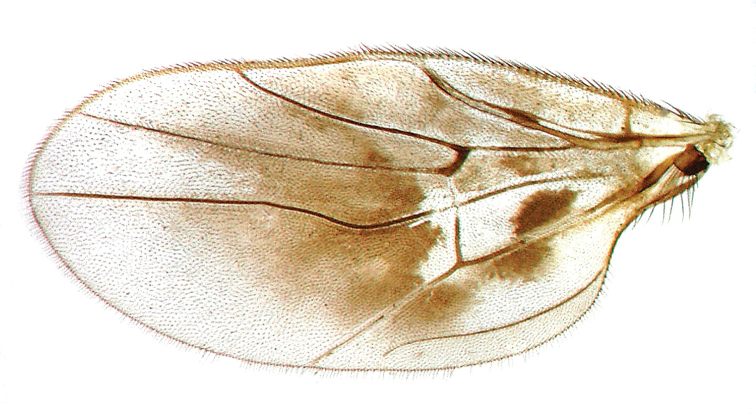
*Megagrapha starki* sp. nov., male wing, paratype.

**Figure 6.**
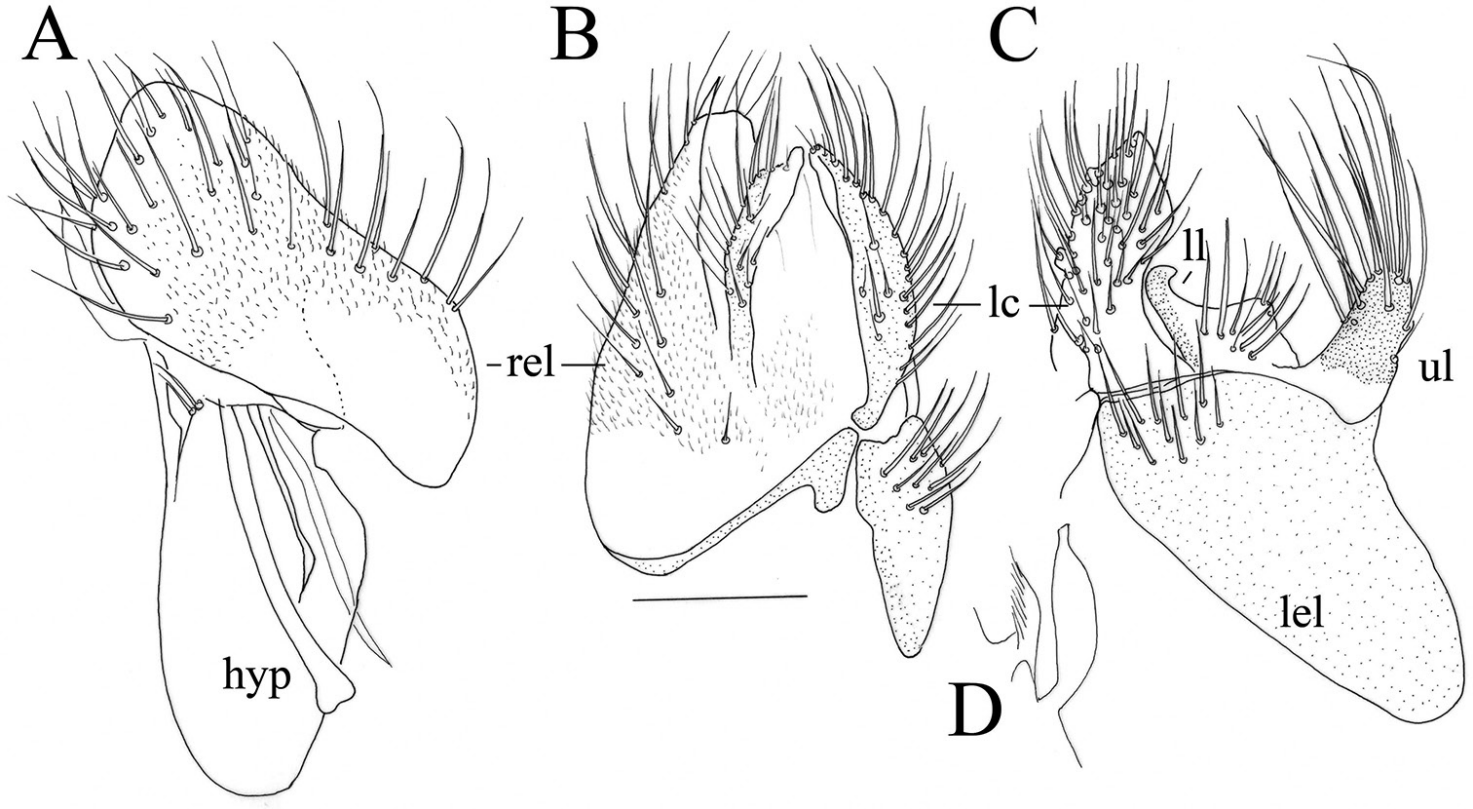
*Megagrapha starki* sp. nov., male terminalia **A** right epandrial lamella **B** epandrium with cerci, dorsal **C** left epandrial lamella **D** detail of phallus. Abbr. hyp: hypandrium; lc: left cercus; lel: left epandrial lamella; ll: lower lobe of left surstylus; ul: upper lobe of left surstylus; rel: right epandrial lamella. Scale bar: 0.1 mm.

A species of *Megagrapha* Melander with patterned wings and modified male fore and mid basitarsi, very similar to Nearctic *M. exquisita* (Malloch, 1923). However, the newly described species differs in the colour of the mesoscutum (Fig. 3), the dark pattern on the wing (Fig. 5), the shape of the postpedicel (Fig. 4) and the male terminalia (Fig. 6).

##### Etymology.

The species epithet, *starki*, is a Latin genitive patronym given in honour of German dipterist Andreas Stark (Halle (Saale)), who first recognised this species as new, acknowledging his contribution to the knowledge of world Empidoidea.

##### Description.

***Male head*** black, rounded in anterior view and ovoid in lateral view. Large eyes with dense and relatively long ommatrichia, all facets subequal in size. Frons very narrow (linear, narrower in lower part than diameter of one ommatidium, widening towards ocellar triangle). Slightly prominent ocellar triangle and adjacent parts of frons and vertex sublustrous, with 2 pairs of short, subequally long and pale ocellars (about 0.10 mm long), with several similar setae posteriorly. Occiput microtrichose (subshiny), with dense yellowish-brown to black setae on dorsal third similar in length to ocellars, ventrally with fewer short, pale setae, posteriorly with several black setae. Face very narrow dorsally and widening ventrally, microtrichose, without setae, gena indistinct. Palpus brown, relatively large (0.22 mm long) and ovoid, slightly narrowing towards tip, covered with relatively long black setae. Labrum yellow, only slightly longer than palpus, labellum narrow with several setae. Antenna yellow (Fig. 4). Scape without setae, 0.08 mm long, pedicel (0.05 mm long) with circlet of short black setae, postpedicel (0.24 mm long) elongated, with rather long narrow apical half. Stylus (0.15 mm long) with several slightly elongated darkened setulae (especially in apical part). ***Thorax*** yellow with brown pattern in form of 2 longitudinal stripes on mesoscutum spreading from black antepronotum posteriorly, bent near scutellum towards postalar calli and connected with a broad stripe medially in front of scutellum (dark pattern in dorsal view roughly U-shaped). Postpronotum and adjacent parts of mesoscutum, including notopleuron, brown, slightly brownish parts occur below wing base including mediotergite. Scutellum black and microtrichose except basolateral corners. Mesoscutum mostly lustrous, pleura shiny except dorsoposterior part of anepisternum. Both anterior and posterior spiracles deep black. Chaetotaxy: antepronotum with 4–5 pale setulae on each side, 3 notopleurals, 1 short postalar, 3 pairs of pale scutellars. Mesoscutum densely covered with black setulae anteriorly (also on postpronotum and adjacent areas) and white setulae posteriorly, setae longer in presutural area with 1 not very prominent prescutellar pair. Pleura with several black setae dorsally on anepisternum. ***Legs*** including coxae very pale (almost whitish-) yellow, rather densely covered with short, whitish setulae, some longer setae only preapically on ventral side of all femora. Basitarsus of fore leg with narrow ventral process and basitarsus of mid leg narrowed basally and widened apically, with short ventral apical projection. Second mid tarsomere with dense setulae ventrally. ***Wing*** (Fig. 5) with distinct dark pattern, covered on both sides with relatively long microtrichia. Basal costal seta only slightly differentiated. Sc incomplete, closely approximated to R_1_ and vanishing at about middle. Costa ends at tip of M_1_ vein. Halter yellow, calypter black with yellow margin and long black fringes. ***Abdomen*** subshiny, with brown (and brown setose) tergites and yellow (pale setose) sternites, lateromarginal setae on tergites as long as or longer than segments. Terminalia as in Fig. 6A–D. Cerci narrow, equally long, shorter than right epandrial lamella. Left surstylus composed of lower lobe (ll) and digitiform upper lobe (ul), bearing setae longer than lobe. ***Length***: body 2.4 mm, wing 2.6 mm.

**Female.** Unknown. However, all allied species are sexually dimorphic with dark-coloured females.

##### Remarks.

Stark (2008) already recognized the species as new and announced a separate paper on *Megagrapha*: “Stark A (2009), in press. Description of two species of the genus *Megagrapha* Melander (Diptera, Empidoidea, Hybotidae, Drapetini) from the canopy of trees in European primeval forests. Studia dipterologica 16(2)”. However, this paper was never published. Andreas Stark kindly sent us illustrations and locality data and his male was included in the type series.

The new species described above is very similar to the Nearctic *M. exquisita* as redescribed by Chillcott and Teskey (1983) and by Chillcott (1958 – male as *M. pubescens* (Loew, 1862)) in the patterned wings and modified basitarsi of the fore and mid legs. However, both species strikingly differ in the colour of the mesoscutum (reddish yellow in *M. exquisita* – ME, with brown stripes in *M. starki* – MS), in the dark pattern on the wing (dark around crossveins of basal cells in ME, light in MS), in the shape of the postpedicel (pear-shaped in ME, with long narrow apical half in MS; Fig. 4) and in the male terminalia (left surstylus longer and more slender in ME than in MS, with bent tip, and apical bristles on upper lobe of digitiform left surstylus shorter than lobe in ME but longer in MS; Fig. 6). *Megagrapha europaea* Papp & Földvári, 2001, the only other known European representative of the genus, has clear wings in the male and mesoscutum yellow without dark stripes. For further differences, see comments under *M. europaea*.

##### Distribution.

Slovakia, Poland, European Russia.

#### Oedalea portugalica

Taxon classificationAnimaliaDipteraHybotidae

Barták & Grootaert sp. nov.

D1D87F7D-7840-56CD-B67F-8C3E95E5EBDA

http://zoobank.org/98C6AF61-ED66-48ED-9C2D-29E337D4C10B

Figs 7 , 8

##### Type material.

***Holotype*** ♂, **Portugal**: 7 km E of Manteigas, nr. river, 580 m, SW, 40°24'42"N, 7°28'4"E, Barták M., 23.v.2008 (CULSP). ***Paratypes***: 3♀, same data as holotype; 1♀, Portugal, 5 km N of Formalicao, Castanea wood, 930 m, SW, 40°28'31"N, 7°21'32"E, Barták M., 23.v.2008 (CULSP).

##### Diagnosis.

**Figure 7.**
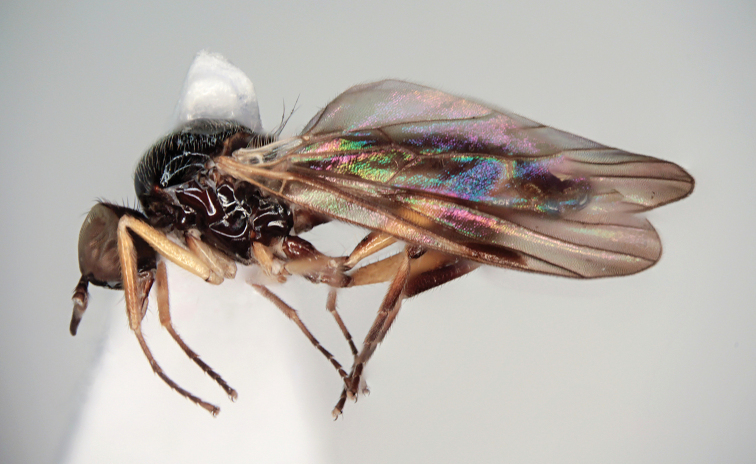
*Oedalea portugalica* sp. nov., male habitus, holotype.

A species of *Oedalea* Meigen with narrow stylus, both black and pale scutellars, conspicuous rather short and wide wing pterostigma, darkened apex of wing, and hind legs yellow with contrastingly dark coxa and trochanter and apical third of femur Fig. 7).

##### Etymology.

The species is named after its country of origin (Portugal).

##### Description.

**Figure 8.**
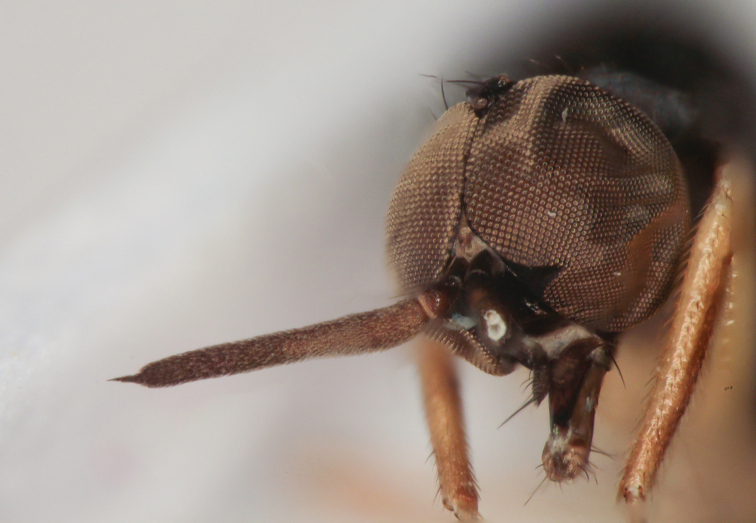
*Oedalea portugalica* sp. nov., male head with antenna (left postpedicel missing), holotype.

***Male head*** black, holoptic. Eyes contiguous over long distance, leaving only a very small frons above antennal base, upper ommatidia much larger than lower. Prominent and lustrous ocellar triangle with pair of black setae (about 0.15 mm long) and additional pair of smaller setae posteriorly. Occiput microtrichose, with black setae on dorsal third forming nearly regular postocular row, laterally more densely setose, ventrally with longer, thin, paler setae. Face lustrous (with slight wrinkles along margins), subequally as long as wide below (0.17 mm), clypeus lustrous, gena very narrow and lustrous. Palpus brown, reaching margin of clypeus, with several dark setae, two apicals somewhat longer. Labrum brown, shiny, directed obliquely, labellum with a few setae. Antenna (Fig. 8) brown, scape and pedicel subequally long (0.08 mm), former without setae, latter with circlet of short setae. Postpedicel very long (almost 0.70 mm), nearly parallel-sided, stylus short and narrow (0.08 mm long). ***Thorax*** black and almost entirely shiny, except following microtrichose parts: prosternum, posterior third of notopleuron (line dividing shiny and microtrichose parts reaches from lower anterior small notopleural seta obliquely dorsally and slightly anterior to long notopleural), area below postalar ridge and mediotergite; propleura with rather long and sparse microtrichia and several pale setae. Scutellum shiny, with distinct wrinkles. Chaetotaxy: prosternum (isolated sclerite) without setae, antepronotum with 5–7 setae on each side, mesoscutum densely covered with short pale setae (those in prescutellar part directed anteriorly), leaving only narrow but distinct bare stripes between acrostichals and dorsocentrals. Single black to pale long notopleural (missing in holotype), 1 short pale postalar, 1 long prescutellar dorsocentral (difficult to observe in holotype due to damage), scutellum with 4 pairs of long setae, outer pairs pale, inner pairs black. Pleura without setae. ***Legs***: fore and mid legs yellow, including coxae and trochanters. Hind coxa and trochanter brown, hind femur yellow in basal part and brown in apical third, hind tibia brown except basal fourth. Basitarsi of all legs yellow to brownish yellow in basal part and darkened apically, remaining tarsomeres brown. Legs covered with short setulae, pale (whitish-yellow to yellow) on proximal parts (coxae, femora, tibiae), dark in more distal parts (some dorsal setae on tibiae and many on tarsi). Hind femur dorsally on basal half with several pale, erect and very long setae (up to 0.25 mm long–about as long as hind tarsomere 2), dark part with usual *Oedalea*-type spines arranged in two irregular rows. ***Wing*** slightly infuscated, markedly so on apical fourth, entirely microtrichose except extreme basal part (proximal to M junction). Basal costal seta pale, only indistinctly differentiated. Sc nearly complete, in apical part closely approximated to R_1_. Costa ends at tip of M_1_ vein, anal vein complete. Halter pale yellow, calypter whitish-yellow with somewhat darker margin and yellow fringes. ***Abdomen*** shiny including genital lamellae, dorsum of tergites with very short pale setae dorsally (in middle of tergites about 0.05 mm long) and much longer laterally (up to 0.20 mm). Sternites shiny, with short pale setae, sternite 1 bare. Some marginal setae on posterior segments darker. Terminalia of holotype not dissected. ***Length***: body 3.9 mm, wing 3.5 mm.

**Female.** Very similar to male except usual sexual differences. Length of postpedicel varies from 0.60 to 0.75 mm. Frons broad and lustrous, bare, about 0.15 mm wide above antenna and 0.25 mm wide at level of front ocellus. Hind coxa and trochanter yellow. Ovipositor relatively short. ***Length***: body 3.2–4.4 mm, wing 3.5–4.4 mm.

##### Remarks.

The new species described above (OP) is very similar to *O. stigmatella* Zetterstedt, 1842 (OS) in having (partly) pale scutellar setae, a narrow stylus and in the colour of the hind femur. However, males of both species differ in the following characters: postpedicel strip-like and at least as long as head height in OP, but distinctly tapered and about 2/3 as long as head height in OS; strong notopleural seta inserted inside microtrichose areas (OP), but on the boundary between shiny and microtrichose areas in OS; wing pterostigma about 3× longer than wide, reaching about half-way between apices of veins R_1_ and R_2+3_ in OP, but about 5× longer than wide, reaching about 2/3 of way between those veins in OS; hind coxa and trochanter brown in OP but yellow in OS. We decided not to dissect the terminalia of the holotype (and only known male) of OP because other characters sufficiently differentiate the new species. Females of OP do not differ from OS in the colour of the hind coxa and trochanters but, besides the other characters given above, have a markedly shorter abdominal segment 8 than OS (about 0.80 mm long incl. cerci in OS vs 0.40 mm long in OP); also, tergite 8 is about as long as its dorsal elongated setae in OP but more than 5× longer in OS. Moreover, all specimens of OP have both black and yellow scutellar setae, whereas these are yellow in OS; however, this character may be variable.

##### Distribution.

Portugal.

#### Platypalpus obscuroides

Taxon classificationAnimaliaDipteraHybotidae

Barták & Grootaert sp. nov.

10CF58CB-DCE9-5873-8F6F-3322AE2B7220

http://zoobank.org/6E21F6BF-255C-4E74-8747-DEEA9EDBB75C

Fig. 9

##### Type material.

***Holotype*** ♂, **Slovakia**: Muráňska planina NPR, Šarkanica, 540 m, MT, 48°42'46"N, 19°59'52"E, M. Tkoč, 6.vi.–6.vii.2018 (CULSP); ***Paratypes***: 1♂, same data as holotype; 3♂, 2♀, same locality, 6.vii.–7.viii.2018 (dissected RBINS); 1♀, same locality, 48°42'46"N, 19°59'56"E, 580 m, 10.viii.–5.x.2017; 2♀, Muráňska planina NP, Hrdzavá dolina, 540 m, MT, 48°44'52"N, 20°01'04"E, J. Roháček, 21.vi.–6.viii.2013 (all CULSP).

##### Diagnosis.

A species of the *Platypalpus albiseta* group with mesonotum and anepisternum entirely microtrichose. Katepisternum with a large shiny spot dorsally, rather stripe-like, reaching from anterior to posterior margins. Meron entirely microtrichose except small subshiny spot ventrally. The new species is similar to *P. obscurus* (von Roser, 1840) except for the much larger male terminalia and the above-mentioned large lustrous spot on the katepisternum.

##### Etymology.

The species name refers to the similarity with *P. obscurus*.

##### Description.

***Male head*** black, entirely grey microtrichose including gena, only clypeus shiny. Frons very narrow (at lower two thirds as wide as anterior ocellus, at narrowest point less than 0.02 mm wide), widening both ventrally (0.06 mm above antennal bases) and dorsally (0.05 mm at level of front ocellus), densely covered with relatively long microtrichia. Slightly prominent ocellar triangle with two pairs of black setae, anterior pair about 0.10 mm long, posterior pair slightly shorter. Occiput microtrichose, subshiny (postocular area with somewhat longer microtrichia giving silvery appearance), with sparse dark setae dorsally and several longer and paler setae ventrally. One pair of long black vertical setae somewhat longer than anterior ocellars and inserted wide apart (0.17 mm). Face very narrow (0.02–0.03 mm), only slightly widening below. Clypeus long, shiny, gena microtrichose. Palpus brown, ovoid and very small (0.07 mm long), with several pale brown setae at tip, one of them longer than palpus. Labrum shiny brown, two thirds as long as head height. Antenna brown with white stylus. Scape without setae, 0.02 mm long; pedicel (0.05 mm long) with circlet of short black setae; postpedicel long (0.32–0.40 mm), narrow (6× longer than wide), with relatively long setulae on both sides, equally narrowing towards tip; stylus half as long as postpedicel (0.16–0.22 mm long), white, dark at extreme base. ***Thorax*** black to brownish black, microtrichose (except lustrous extreme anterior part of mesoscutum and part of katepisternum), in anterior view with broad velvety-brown stripe below acrostichals and in posterior view with two silvery lines between acrostichals and dorsocentrals. Katepisternum with large shiny spot dorsally, rather stripe-like, reaching from anterior to posterior margins, lower half microtrichose. Meron entirely microtrichose except small subshiny spot ventrally. Chaetotaxy: antepronotum with several short pale setulae on each side, postpronotum with scarcely differentiated setae, 1 notopleural, 1 short postalar. Acrostichals inclinate, in 6–8 irregular rows, only indistinctly separated from nearly uniserial dorsocentrals, both short (about 0.07 mm long), one prescutellar seta; 2 pairs of scutellars, outer pair much shorter.

**Figure 9.**
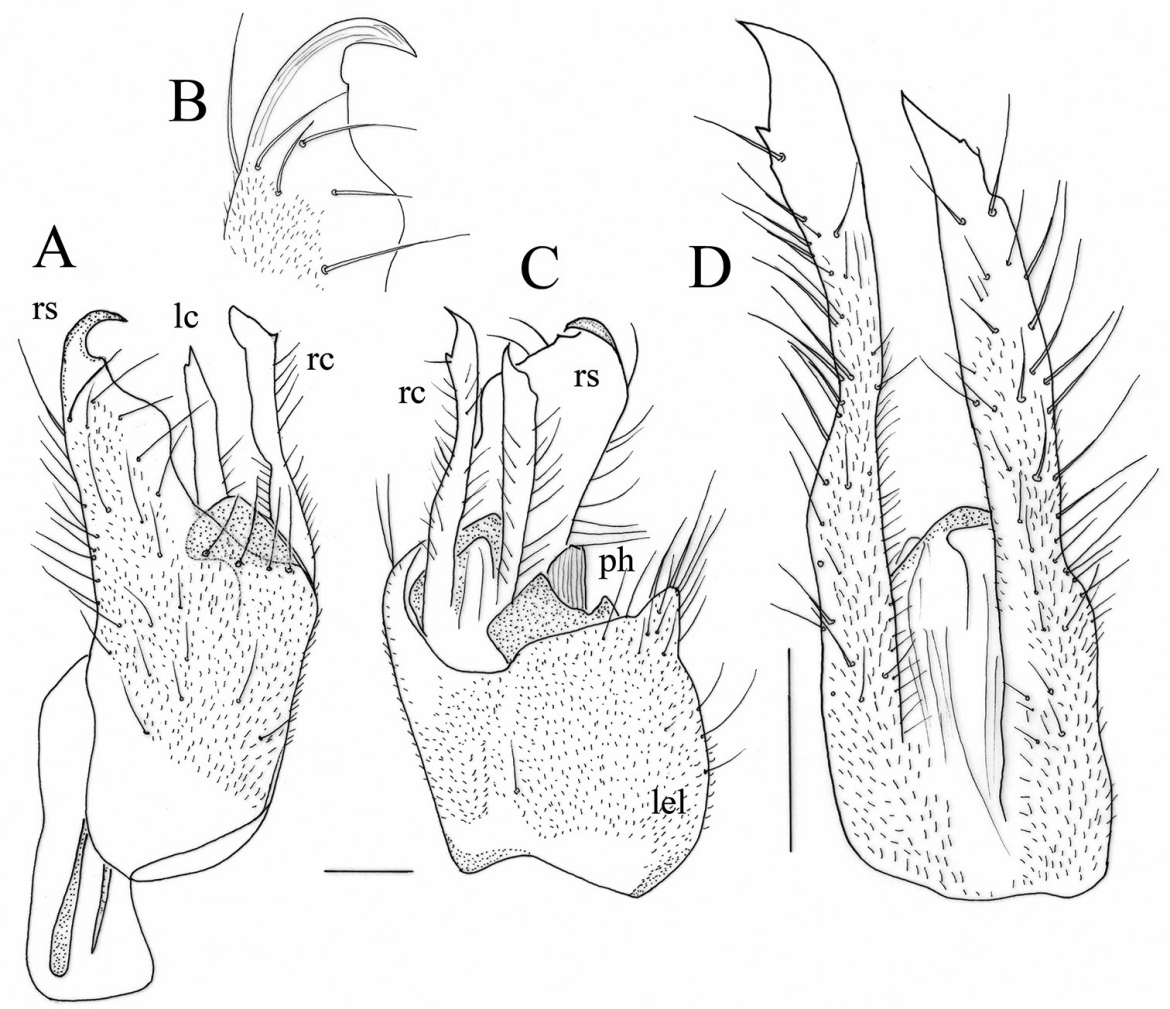
*Platypalpus obscuroides* sp. nov., male terminalia **A** epandrium, from right side **B** apex of right epandrial lamella **C** epandrium and cerci, dorsolateral view **D** cerci, dorsal view. Abbr. lc: left cercus; lel: left epandrial lamella; ph: phallus; rc: right cercus; rs: right surstylus. Scale bars: 0.1 mm.

***Legs*** including coxae brown to black, fore coxa and base of fore femur more or less yellowish, some specimens with paler mid coxa or basal part of mid and hind femora. Setae both pale and brown. Fore femur with two ventral rows of setae (somewhat golden-brown under some angles of view) almost as long as diameter of femur. Fore tibia strongly spindle-shaped, dilated (at broadest point twice wider than before apex), densely set with ventral pubescence as long as tibia depth. Mid femur nearly as wide as fore femur, slightly swollen in basal quarter only, distal part thin, with anteroventral row of black spinules, long about base, with several anteroventral setae similarly longer about base, posteroventral spines somewhat longer than anteroventral spines; short dense white pubescence between anterior and posterior rows of spinules; 4–6 dark posteroventral setae as long as femur depth, inserted almost in same line as spines. Hind femur nearly as wide as mid femur, with ventral row of pale setulae about as long as femur depth. Hind tibia ventrally with whitish setulae as long as tibia depth. ***Wing*** brown-infuscated especially in anterior half, entirely microtrichose. Basal costal seta brown, long. Subcosta incomplete, ending free; crossveins contiguous; CuA_2_ strongly recurrent; anal vein distinct throughout; vein M_1_ slightly S-shaped; C ends at tip of M_1_ vein. Halter brown, calypter black in proximal part and brownish-yellow in distal half, fringe pale. ***Abdomen*** brown, genital lamellae contrastingly black. Tergites thinly microtrichose, subshiny, sternites microtrichose. Setae mostly pale, short, those on last segment longer. Terminalia as in Fig. 9A–D, very large (total length nearly 0.70 mm). Apices of both cerci pointed. Right epandrial lamella with single, acute, bent tip (Fig. 9A). Apex of left epandrial lamella rather shorter than in *P. obscurus*. ***Length***: body 2.4–2.7 mm, wing 2.5–3.3 mm.

**Female.** Very similar to male in all details except the following: abdomen more microtrichose and segment 8 very long (about 0.60 mm long including ventral prolongation, longer than four preceding segments combined), shiny black at side, narrow dorsal stripe not sclerotized. ***Length***: body 2.9–3.5 mm, wing 2.7–3.0 mm.

##### Remarks.

**Figure 10.**
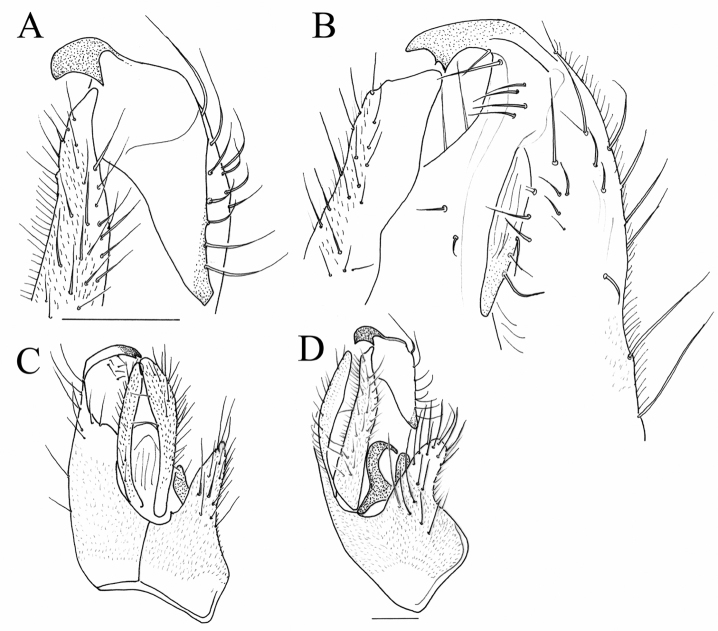
*Platypalpus obscurus* (von Roser), male terminalia **A** dorsal view of apex of right epandrial lamella and left cercus **B** lateral view of apex of right epandrial lamella **C** epandrium, dorsal **D** epandrium, left lateral. Scale bars: 0.1 mm.

*Platypalpus obscuroides* (POI) is very similar to *P. obscurus* (POS). However, it differs in the length of the stylus, which in the new species (POI) is about half as long as the postpedicel, while in POS it is nearly as long as the postpedicel. Further differences are: katepisternum in POI widely lustrous up to its hind margin, whereas with only a small oval lustrous patch not reaching hind margin of katepisternum and narrowly lustrous posterior margin in POS. Male terminalia: left cercus wider and as long as right cercus in POS (Fig. 10C), versus both cerci pointed and right cercus longer in POI (Fig. 9D); apex of right epandrial lamella bent in POI (Fig. 9A), versus as two claw-like projections in POS (Fig. 10D). The female of POI has a shiny abdominal segment 8, while it is almost entirely microtrichose in POS.

##### Distribution.

Slovakia.

### Key to European species of the *P. obscurus* complex

Species of the *P. albiseta* group with both anepisternum and mesoscutum microtrichose.

**Table:** 

1	Fore tibia strongly spindle-shaped, dilated. Female abdominal segment 8 conspicuously elongated	**2**
–	Fore tibia narrow, not dilated. Female abdominal segment 8 short	**3**
2 (1)	Katepisternum with lustrous spot reaching posterior margin. Female abdominal segment 8 shiny. Male right cercus bent at tip, forming lustrous flap (Fig. 9A, B)	***P. obscuroides* sp. nov.**
–	Katepisternum with small lustrous spot not connected with narrow lustrous posterior margin. Female abdominal segment 8 microtrichose. Male right cercus simply rounded and microtrichose at tip (Fig. 10D)	***P. obscurus* (von Roser)**
3 (1)	Postpedicel at most twice longer than wide, stylus much longer than postpedicel. Legs completely dark	***P. argentiseta* (Collin)**
–	Not as above	**4**
4 (3)	Stylus shorter than postpedicel. Acrostichals biserial. Katepisternum with small lustrous patch not reaching posterior margin. Legs black	***P. albistylus* Chvála**
–	Stylus longer than postpedicel. Acrostichals 6–8-serial. Katepisternum with large lustrous patch reaching posterior margin. Legs partly yellow	***P. pallidiseta* Kovalev**

### Redescriptions and faunistic records

#### Syndyas merzi

Taxon classificationAnimaliaDipteraHybotidae

Shamshev & Grootaert, 2012

23235F97-484D-54FF-AC05-D17EBB3055A8

##### Material examined.

**Turkey.** 2♂, 2 km W of Toparlar, lowland wood, 36°59'27"N, 28°38'49"E, Barták, Kubík, 18.ix.2019; 1♀, Toparlar, lowland wood, 8 m, 36°59'27"N, 28°38'50"E, Barták, Kubík, 22–24.vi.2015; 1♀, same locality, 11.ix.2014 (all CULSP). First records from Turkey.

##### First description of female

(only characters different from male or not specified in original description are given). ***Head***: postpedicel 0.12 mm long and 0.06 mm wide, stylus about 0.35 mm long and virtually bare, apical seta-like mechanoreceptor difficult to distinguish but seems ca. 0.05 mm long; labrum 0.40 mm long (as long as length of compound eye); eyes contiguous below antennae. ***Thorax***: mesoscutum lustrous, including postpronotal lobes, posterior part in front of scutellum and lateral parts of notopleuron microtrichose. Setae pale in anterior part and dark in posterior part. 3–4 pairs of long, irregularly-arranged prescutellar setae, 3 pairs of scutellars, outer pair shortest and central part longest. Acrostichals moderately long and irregularly 6-serial anteriorly, much shorter, darker, almost biserial posteriorly (in male, anteriorly also 6-serial but very short or absent posteriorly). Pleura entirely microtrichose. ***Legs***: fore tibia with scattered posteroventrals slightly longer than tibia depth. Ventral preapical seta on mid tibia about 0.20 mm long and similar seta also on mid basitarsus. Hind femur, beside strong anteroventrals, with rows of fine posteroventrals and posterodorsals subequally long as femur depth. Hind tibia with 3 strong anterodorsals. ***Abdomen***: tergites 2–7 lustrous, tergites 1 and 8 subshiny, sternites 1–6 lustrous, sternite 7 subshiny and sternite 8 microtrichose, sternite 1 bare. Tergites with short setae dorsally and longer setae laterally (lateromarginals almost as long as tergites), sternites sparsely setose (with some 4 pairs of rather long setae on each sternite). ***Length*** of body 3.1 mm, of wing 2.5 mm.

##### Remarks.

Male abdominal tergites shiny except dorsal part of tergites 1–3 and base of tergite 4. Our specimens are slightly smaller than stated in original description.

##### Distribution.

Cyprus, Turkey.

#### Megagrapha europaea

Taxon classificationAnimaliaDipteraHybotidae

Papp & Földvári, 2001

BAF07A61-A45A-59B2-9EF9-362778A451E9

Fig. 11

##### Material examined.

**Slovakia.** 3♂, 1♀, Muráňska planina NPR, Šarkanica, 580 m, MT, 48°42'46"N, 19°59'56"E, J. Roháček 14.vi.–11.vii. 2017 (2♂, 1♀ CULSP, 1♂ RBINS); 1♀, Muráňska planina NP, Tisovec, 610 m, MT, 48°42'07"N, 19°58'58"E, M.Tkoč 20.v.–18.vi.2019 (RBINS). First records from Slovakia.

##### Redescription.

**Figure 11.**
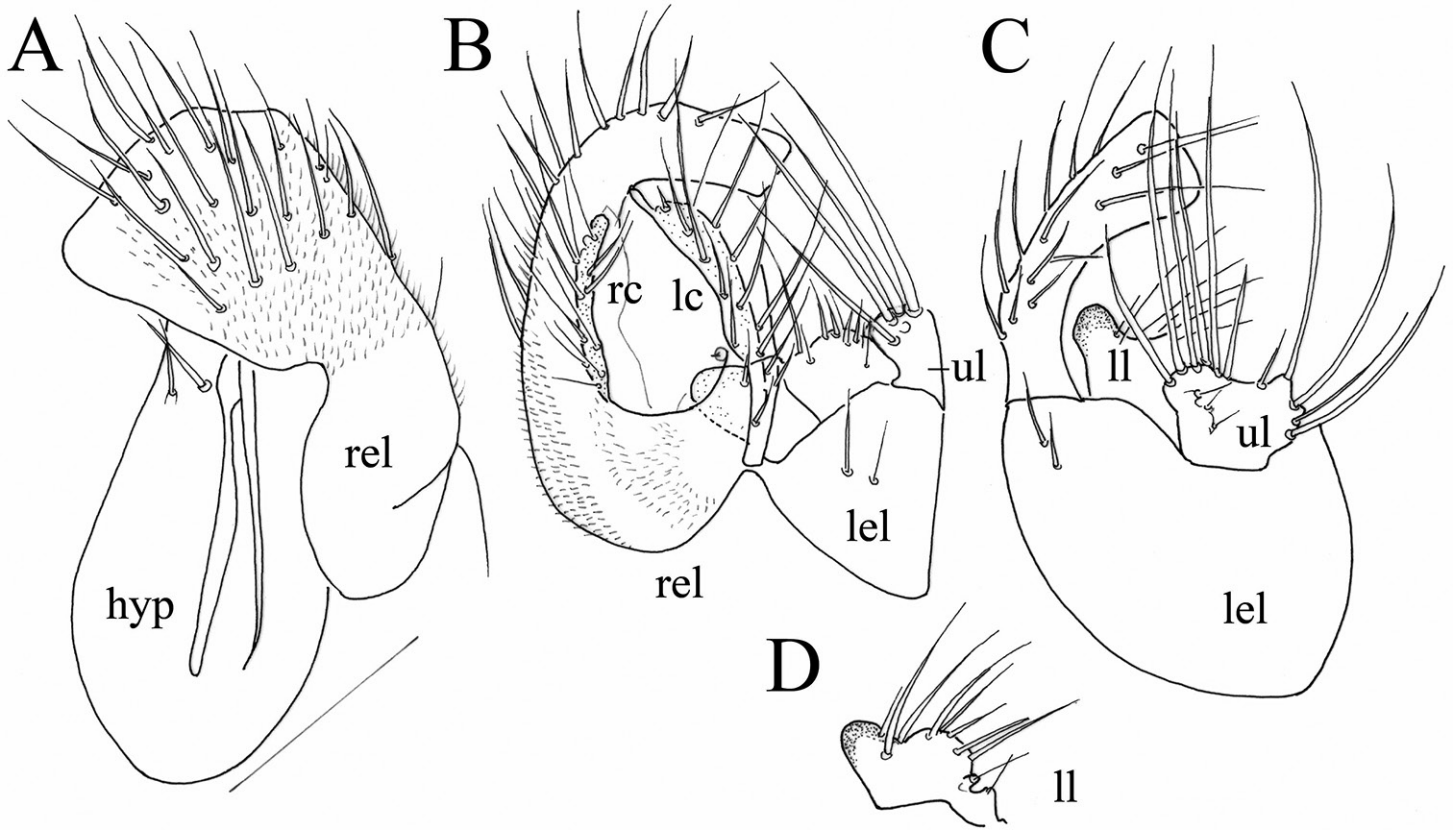
*Megagrapha europaea*, male terminalia **A** right epandrial lamella **B** epandrium with cerci, dorsal view **C** left epandrial lamella **D** lower lobe of left surstylus. Abbr. hyp: hypandrium; lc: left cercus; lel: left epandrial lamella; ll: lower lobe of left surstylus; rc: right cercus; rel: right epandrial lamella; ul: upper lobe of left surstylus. Scale bar: 0.1 mm.

This species strongly resembles *M. thaica* Grootaert & Shamshev, 2009, except for the following features. Postpedicel brown in contrast to yellow pedicel. Mid tarsomere 1 about 2.5 times as long as mid tarsomere 2. Terminalia (Fig. 11) very small, brownish yellow, rather subglobular. Cerci separated; right cercus digitiform, slightly tapered, with several long unmodified setae; left cercus longer and stronger than right cercus, digitiform when viewed dorsally, somewhat narrowed subapically, subrectangular when viewed laterally, with several long unmodified setae. Epandrium completely divided. Right epandrial lamella subrectangular (viewed laterally), with numerous long setae on apical half. Right surstylus not prominent. Left epandrial lamella separated from hypandrium, with 1 short seta apically. Left surstylus differentiated from epandrium, represented by two elements; upper lobe bilobed, with cluster of long bristles on one lobe, two long bristles on second lobe; lower lobe strongly sclerotised, with cluster of shorter, spine-like setae. Hypandrium with 3 short setae apically. Phallus very short. Ejaculatory and ventral apodemes subequally long.

##### Remarks.

The male terminalia of *M. starki* sp. nov. are similar to those of *M. europaea*, but different in many details. The most obvious difference is that the upper lobe of the left surstylus is short and bifid in *M. europaea*, but long digitiform in *M. starki* sp. nov.

##### Distribution.

Hungary, Slovakia.

## Supplementary Material

XML Treatment for Hybos conicus

XML Treatment for Megagrapha starki

XML Treatment for Oedalea portugalica

XML Treatment for Platypalpus obscuroides

XML Treatment for Syndyas merzi

XML Treatment for Megagrapha europaea
